# Recurrent neural network for predicting absence of heterozygosity from low pass WGS with ultra-low depth

**DOI:** 10.1186/s12864-024-10400-4

**Published:** 2024-05-14

**Authors:** Fei Tang, Zhonghua Wang, Yan Sun, Linlin Fan, Yun Yang, Xueqin Guo, Yaoshen Wang, Saiying Yan, Zhihong Qiao, Yun Li, Ting Jiang, Xiaoli Wang, Jianfen Man, Lina Wang, Shunyao Wang, Huanhuan Peng, Zhiyu Peng, Xiaoyuan Xie, Lijie Song

**Affiliations:** 1 Clin Lab, BGI Genomics, Tianjin, 300308 China; 2 BGI Genomics, Shenzhen, 518083 China; 3 Clin Lab, BGI Genomics, Wuhan, 430074 China; 4 Clin Lab, BGI Genomics, Shenzhen, 518083 China; 5 Tianjin Women’s and Children’s Health Center, Tianjin, 300070 China; 6 DTU Bioengineering, Technical University of Denmark, 2800 Kongens Lyngby, Denmark

**Keywords:** AOH, RNN, LP-WGS

## Abstract

**Background:**

The absence of heterozygosity (AOH) is a kind of genomic change characterized by a long contiguous region of homozygous alleles in a chromosome, which may cause human genetic disorders. However, no method of low-pass whole genome sequencing (LP-WGS) has been reported for the detection of AOH in a low-pass setting of less than onefold. We developed a method, termed CNVseq-AOH, for predicting the absence of heterozygosity using LP-WGS with ultra-low sequencing data, which overcomes the sparse nature of typical LP-WGS data by combing population-based haplotype information, adjustable sliding windows, and recurrent neural network (RNN). We tested the feasibility of CNVseq-AOH for the detection of AOH in 409 cases (11 AOH regions for model training and 863 AOH regions for validation) from the 1000 Genomes Project (1KGP). AOH detection using CNVseq-AOH was also performed on 6 clinical cases with previously ascertained AOHs by whole exome sequencing (WES).

**Results:**

Using SNP-based microarray results as reference (AOHs detected by CNVseq-AOH with at least a 50% overlap with the AOHs detected by chromosomal microarray analysis), 409 samples (863 AOH regions) in the 1KGP were used for concordant analysis. For 784 AOHs on autosomes and 79 AOHs on the X chromosome, CNVseq-AOH can predict AOHs with a concordant rate of 96.23% and 59.49% respectively based on the analysis of 0.1-fold LP-WGS data, which is far lower than the current standard in the field. Using 0.1-fold LP-WGS data, CNVseq-AOH revealed 5 additional AOHs (larger than 10 Mb in size) in the 409 samples. We further analyzed AOHs larger than 10 Mb, which is recommended for reporting the possibility of UPD. For the 291 AOH regions larger than 10 Mb, CNVseq-AOH can predict AOHs with a concordant rate of 99.66% with only 0.1-fold LP-WGS data. In the 6 clinical cases, CNVseq-AOH revealed all 15 known AOH regions.

**Conclusions:**

Here we reported a method for analyzing LP-WGS data to accurately identify regions of AOH, which possesses great potential to improve genetic testing of AOH.

**Supplementary Information:**

The online version contains supplementary material available at 10.1186/s12864-024-10400-4.

## Background

The absence of heterozygosity (AOH) is a kind of genomic change characterized by a long contiguous region of homozygous alleles in a chromosome [1]. Several underlying mechanisms of AOH have been reported, such as meiotic segregation errors [2], parental consanguinity [3], or complex chromosomal rearrangements [4]. AOHs do not necessarily have clinical consequences, however, they may cause serious pathogenic effects when it is related to imprinting effects [5] or autosomal recessive disease mechanisms [3]. For example, more than 25% of patients with Prader–Willi syndrome are caused by isodisomy (the inheritance of both homologs from a single parent and only one homolog of that parent is present) or heterodisomy (the inheritance of both homologs from a single parent and both homologs of that parent are present) [6]. Sahoo et al*.* found that whole-genome uniparental isodisomy (UPD) caused pregnancy loss in ~ 1% of cases [7]. In a study of rare autosomal trisomy by genome-wide noninvasive prenatal testing, the author found that 4.16% of cases with rare autosomal trisomies originate from uniparental disomy [8]. All these studies illustrated the significance of AOH detection for the diagnosis of specific imprinting disorders and rare Mendelian diseases caused by homozygosity.

In the past, short tandem repeat (STR) testing and methylation analysis were the most commonly used methods for the detection of AOH. In recent years, chromosomal microarray analysis (CMA) has been recommended for the detection of AOH in clinical laboratories [9]. CMA can detect long continuous regions of AOH by examining the allele patterns across a chromosome. However, due to the probe density, the detection resolution varied a lot across different platforms and probes. Currently, low pass whole genome sequencing (LP-WGS), a massive parallel sequencing (MPS) based technology, has been widely used in clinical settings for its superiority in the detection of copy-number variants (CNVs) [6, 9, 10]. Studies detecting AOH were based on the analysis of B-allele frequencies, which required a relatively high sequencing depth to obtain variant allele fraction. Recently, one study reported the detection of AOH using LP-WGS with a read depth of ~ fourfold [11]. As is known, most contemporary implementations of LP-WGS are based on a read depth of ~ 0.25, which is the current standard of LP-WGS for CNV detection in the field [6, 9, 10]. To the best of our knowledge, no method of LP-WGS has been reported for the detection of AOH in a low-pass setting of less than onefold.

In this study, we described a recurrent neural network (RNN) based method for predicting AOH using LP-WGS, which we termed as CNVseq-AOH. This method overcomes the sparse nature of typical LP-WGS data by combining population-based haplotype information, adjustable sliding windows, and RNN. It can distinguish AOH regions based on analysis of ~ 0.1-fold low-coverage whole-genome sequencing data, which is far lower than the currently used sequencing depth in the field. To validate the feasibility of this method for AOH detection, we further tested CNVseq-AOH using 409 cases (high coverage WGS data obtained from the 1000 Genomes Project (1KGP)) with previously ascertained CMA results of varying sequencing depths and 6 clinical cases. The results showed that our method possesses great potential to improve the genetic testing of AOH. The source code is available on GitHub with a free license for noncommercial use (https://github.com/helplessness/CNVseq-AOH).

## Methods

### 409 cases with known AOH in the 1KGP

In the 1KGP, there are 413 cases with previously identified AOH events based on SNP-based microarrays and high coverage WGS data (https://www.ebi.ac.uk/ena/browser/view/PRJEB31736?show=reads). In this study, we selected 409 cases (excluding 4 cases with mosaic AOH) for the testing of our method. These 409 samples encompass multiple ethnicities, including multiple ethnicities such as EUR, SAS, AFR and so on. For model testing, we randomly selected 11 cases (11 AOH regions) for training our method and the rest cases for validation, with no overlap, to obtain the optimal RNN architecture.

### LP-WGS for 6 clinical cases

To test the performance of CNVseq-AOH in a real clinical setting, a total of 6 clinical cases (15 ascertained AOH regions) were recruited. All the 15 AOHs were confirmed previously by WES. 50 ng DNA was used for library preparation. LP-WGS (single-end, 35 bp) was performed on the MGISEQ-2000 platform and analyzed as previously described [12]. Informed consent for the anonymous usage of remaining samples and data for scientific research and possible publication was obtained from all participants. This study was approved by THE INSTITUTIONAL REVIEW BOARD OF BGI (NO. BGI-IRB 22062).

### CNVseq-AOH

**Fig. 1:**
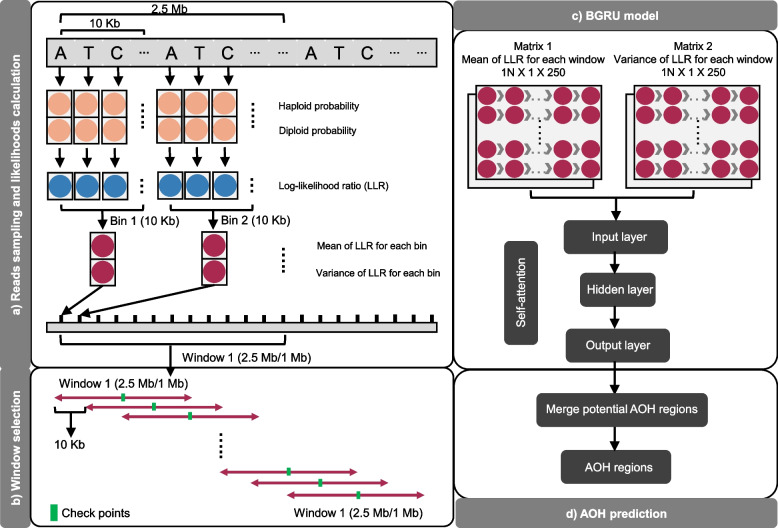
The structure of CNVseq-AOH

Python was used for the development of CNVseq-AOH. CNVseq-AOH aimed to predict AOHs by using the information extracted by RNN from LP-WGS data. RNN was widely used in natural language processing as it is effective at processing sequential inputs. In this paper, DNA sequence was considered as a sentence, while k-mer was considered as a word. First, CNVseq-AOH performed sampling for aligned reads and calculated the probabilities of possible haplotypes based on population-derived haplotype information. Concurrently, for all single nucleotide polymorphisms (SNPs), the LLR (log-likelihood ratio) between haploids and diploids was calculated. Subsequently, the average and variance of the LLR for SNPs within the designated bin were computed. Thirdly, we used a sliding window of size N to scan all the bins to construct an N-dimensional vector that represented the context of the center SNP. Finally, these vectors were deposited into a matrix. A Bidirectional Gated Recurrent Unit (BGRU) was then used for feature learning and classification, and to predict AOHs. CNVseq-AOH mainly consists of the following steps (Fig. 1):

### Alignment, reads sampling, and likelihoods calculation

For data in the FASTQ format, raw sequencing data is aligned using BWA (version 0.7.17-r1188) to obtain clean reads [13]. To overcome the sparse nature of low-coverage sequencing data, we calculated the probabilities of possible haplotypes for resampled reads based on the linkage disequilibrium (LD) measured in a matched population reference [14]. The LD contains population-based haplotype information matched to the ancestry of the testing samples. The introduction of LD of our method for probability calculation is based on the premise that the probability of drawing reads from the same haplotype differs under different ploidy hypotheses [14]. The probabilities were calculated as follows:

First, the DNA sequence was divided into bins with a size of 10 Kb. The information of all biallelic SNPs that overlap with the read in each bin is extracted according to an ancestry-matched reference panel from 1KGP. Then, possible haplotypes (haploid and diploid) and their frequencies would be listed and calculated respectively based on the previously extracted information. The probabilities for every possible haplotype can reflect the number of common haplotypes existing in the population in the chromosomal region that overlaps with the read. Then, the LLR was computed for each SNP. Finally, the mean and variance of LLR for each bin were calculated.

### Window selection

The 409 samples were randomly down-sampled to five different depths (0.05-fold, 0.1-fold, 0.5-fold, onefold, threefold) for analysis. A defined window (250 bins for 0.5-fold, onefold, and threefold depth; 100 bins for 0.05-fold and 0.1-fold depth) with 10 Kb increment in the genome was used to calculate the statistics. For each sliding window, the mean and variance of all bins (250 or 100) in the window were calculated. The bins with no data were skipped and the next valid bin was included to ensure sufficient bins (250 or 100) of each sliding window for calculation. The central coordinates for each sliding window were utilized as check points, and the state of whether the checkpoint was located in an AOH region was employed to represent the window region.

### Feature learning and classification using RNN

RNN is especially useful with sequential data. The structure of our RNN model is shown in Fig. 1. The sequential data (matrix 1: mean of LLR for each window; matrix 2: variance of LLR for each window) obtained in the previous step was then sent into the BGRU network for feature learning and classification.

#### BGRU

To capture complex context information across the genome and balance between the previous memory state and the new candidate memory state. BGRU was implemented, which was achieved through the following formulas:

The following equation is used to compute the update gate Z_t_:

$$\overleftarrow{{Z}_{t}}=\sigma ({W}_{xz}{X}_{t}+{W}_{hz}\overleftarrow{{H}_{t-1}}+{b}_{z})$$

where Z_t_ is the activation value of the update gate. σ indicates the activation function. W_xz_ and W_hz_ represent the input state and hidden state of the weight matrices respectively. X_t_ indicates the input vector. H_t-1_ represents the previous hidden state. b_z_ indicates biases.

The following equation characterizes the reset gate R_t_.

$$\overleftarrow{{R}_{t}}=\sigma ({W}_{xr}{X}_{t}+{W}_{hr}\overleftarrow{{H}_{t-1}}+{b}_{r})$$

The following equation is used to compute the hidden state N_t_, where $\otimes$  indicates the element-wise multiplication.

$$\overleftarrow{N_t}=tanh(W_{xn}X_t+W_{hn}(\overleftarrow{R_t}\otimes\overleftarrow{H_{t-1}})+b_n)$$

The following equation is used to compute the memory state H_t_:

$$\overleftarrow{H_t}=\left(1-\overleftarrow{Z_t}\right)\overleftarrow{N_t}+\overleftarrow{Z_t}\otimes\overleftarrow{H_{t-1}}$$

The following equation is used to compute the output H_t_, which also showed the forward and backward of the memory state respectively.

$${H}_{t}=merge(\overrightarrow{{H}_{t}},\overleftarrow{{H}_{t}} )$$

During this process, self-attention was implemented for the matrix. Through a hidden layer of 64 neurons, the state of whether each checkpoint is located in an AOH region (0–1) is obtained as the output layer.

### AOH prediction

The log-likelihood ratio over larger genomic intervals was summed up to predict AOHs across the genome. In short, the first continuous regions with the log-likelihood value of more than 0.6 were considered as potential AOH regions. Adjacent potential AOH regions will then be merged. After removing N regions in the genome, prediction results for the whole genome will be obtained.

### Depth evaluation

For the 409 samples with positive CMA results in the 1KGP, down-sampling samples were randomly down-sampled to a certain sequencing depth using DownsampleSam (Picard). For each down-sampling sample, true positives detected by a certain depth were classified as AOH regions with at least a 50% overlap with CMA-detected AOH regions and confirmed by visualization using an in-house script. Potential inconsistent results between CNVseq-AOH and CMA were further confirmed by visualization of the SNP ratio using the original high read depth LP-WGS data.

## Results

### Performance of CNVseq-AOH on the 1KGP

The average sequencing depth of the 409 cases with previously identified AOH events from 1 KG was ~ 30-fold. Using high-coverage WGS data, 409 samples with positive CMA results were randomly down-sampled to five different depths (0.05-fold, 0.1-fold, 0.5-fold, onefold, threefold) for analysis.

Ancestry-matched population (or genetically similar population) was used for analysis. A total of 11 randomly selected cases from the 1KGP were used for training our method. For samples with a depth of 0.5-fold to threefold, a 2.5 Mb-window with 10 kb increments was used to train CNVseq-AOH on the training set and tested it on the validation set respectively. For samples with ultra-low depths of 0.05-fold and 0.1-fold, a different model (1 Mb-window with 10 kb increments) was performed to improve detection sensitivity. A 588,112 X 2 X 250 binary matrix was generated for the training datasets. A fixed of 300 steps and early stopping (99.5% accuracy) were set for the training set. The model with the highest accuracy before early stopping will be selected. For the validation set, all test results were listed in Supplementary Table 1.

There were a total of 863 previously identified AOH events in the 409 cases, including 784 AOHs on autosomes and 79 AOHs on the X chromosome. In phase three of the 1KGP, variants on autosomes were phased by SHAPEIT2 (statistical phasing with pedigree-based correction) [15], while variants on the X chromosome were phased by Eagle2 (without the pedigree-based correction) [16]. Due to this inconsistency in variant phasing, the probability calculation of CNVseq-AOH may be influenced. So, we separately calculated the concordant rate on autosomes and the X chromosome.

**Fig. 2:**
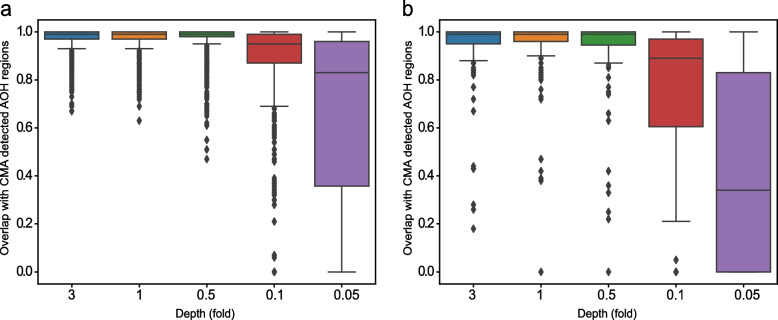
Performance of CNVseq-AOH for 784 AOHs on autosome (**a**) and 79 AOHs on X chromosome (**b**) respectively

For the 784 AOHs on autosomes, in general, the prediction sensitivity of CNVseq-AOH increased with depth (Fig. 2a). As expected, the sensitivity of CNVseq-AOH was 100% (784/784) when the depth was >  = onefold (Supplementary Table 1). With a depth of 0.5-fold, the sensitivity reached 99.9%. Only one AOH with an overlap of 47% was missed by CNVseq-AOH (Supplementary Table 1). The sensitivity of CNVseq-AOH reached 96.23% even with a depth of 0.1-fold, which is far lower than current studies, which need 4-to-fivefold depth [11, 17]. For the 79 AOHs on the X chromosome, the sensitivity of CNVseq-AOH was 59.49% (47/79) with a depth of 0.1-fold. The prediction sensitivity of CNVseq-AOH also increased with depth (Fig. 2b). However, even with a depth of threefold, the prediction sensitivity is still not 100%. There were 6 AOHs missed by CNVseq-AOH with an overlap ranging from 18%-44%. These 6 AOHs were located in similar regions on the X chromosome (Supplementary Table 1), which were also missed by CNVseq-AOH when using 0.5-fold and onefold depth. We further calculated the SNP numbers per 1 Mb on all the chromosomes in the 1KGP. The number of SNPs per 1 Mb on the X chromosome (mean of 26,839.9) was significantly less than the number of autosomes (18,439.9) (T-test, with P-value of 2.94E-12). One reasonable explanation for the relatively low sensitivity for AOHs on the X chromosome is that, compared with autosome, the variant information in the phasing results of the X chromosome in 1KGP was insufficient to calculate the probabilities for resampled reads.

For SNP-based microarrays, a threshold of >  = 10 Mb has been suggested for reporting AOH [18]. In the real clinical setting, AOH larger than 10 Mb in one chromosome is recommended for reporting the possibility of UPD [19, 20]. There were 291 AOH regions larger than 10 Mb in the 1KGP. For these AOHs, CNVseq-AOH can predict AOHs with a sensitivity of 100% (291/291) when the depth was >  = 0.5-fold (Supplementary Table 1). With a depth of 0.1-fold, the sensitivity reached 99.66% (290/291). CNVseq-AOH provided a prediction sensitivity of 94.5% (275/291) even with a depth of 0.05-fold.

For 0.1-fold LP-WGS data, it takes an average of 11 min to process a single sample using an 8-core CPU with 8 GB of RAM (from data alignment to reporting), including an average of 10 min for alignment, 25 s for feature learning, and 10 s for AOH prediction and reporting.

### Additional AOHs detected by CNVseq-AOH

Compared to AOHs detected by CMA, additional AOHs were detected by CNVseq-AOH. We analyzed additional AOHs detected by CNVseq-AOH with a depth of 0.1-fold. A total of 267 additional AOHs were detected in the 409 samples by CNVseq-AOH, approximately 0.65 AOHs for each sample. The number of the additionally detected AOHs decreased with the length of AOH (Supplementary Fig. 1). Using high-coverage data, we further validated these AOHs by visualization using an in-house script. The results showed that, 50.56% (135/267) additional AOHs were true positives (Supplementary Table 2; Supplementary Fig. 2). In the clinical setting, a threshold of > 10 Mb was recommended for reporting the possibility of UPD [19, 20]. Using a threshold of > 10 Mb, only 5 additional AOHs were detected by CNVseq-AOH for the 409 samples with 0.1-fold depth (Supplementary Table 2).

**Fig. 3:**
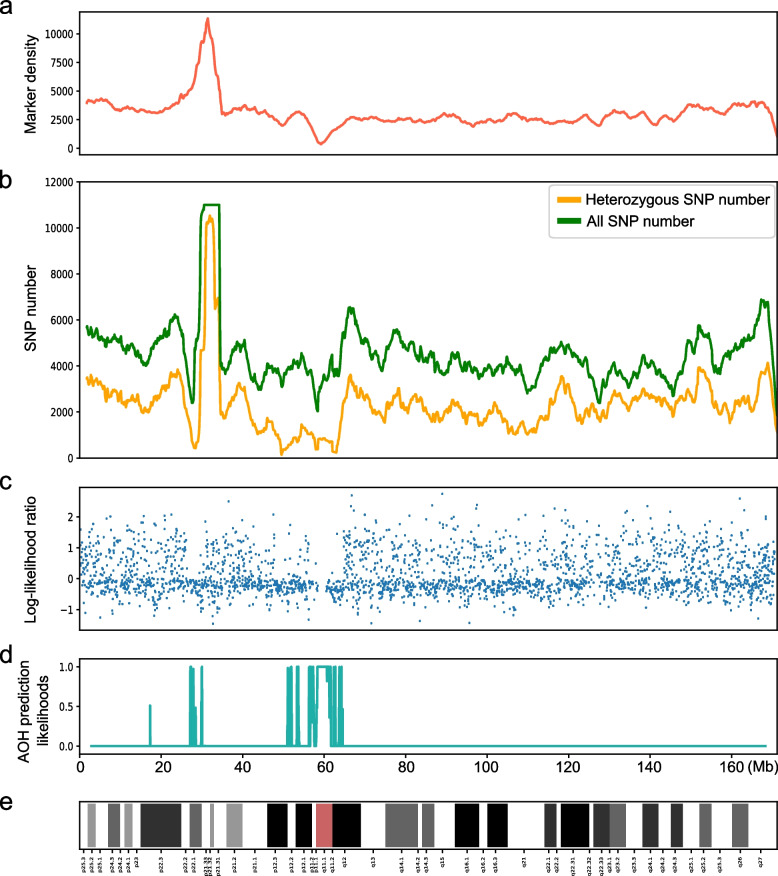
AOH region (seq[GRCh38] hmz(6)(p12.3q12) chr6:g. 47568317_64568317hmz) detected by CNVseq-AOH in chromosome 6 of HG01980. **A** Marker of HumanOmni2.5–4 (SNP-based microarray kit used in the 1KGP) in chromosome 6. Marker density in this region of the sample represents sufficient markers for the chr6:g. 47568317_64568317 region. However, no AOH was reported in this region by CMA, which indirectly reflects the detection performance of CNVseq-AOH for regions crossing the centromeric regions; **b** The number of heterozygous SNP number (yellow line) and all SNP number (green line) in this region calculated using high coverage WGS data. The heterozygous SNP number (yellow line) in the chr6:g. 47568317_64568317 region was close to 0, which indicated potential AOH events in this region; **c** Log-likelihood ratio for haploid and diploid in each bin. Each dot represents the mean log-likelihood ratio in each bin. For potential AOH regions, the log-likelihood ratio tends to approach 0, with relatively sparse blue dots above 0; **d** AOH prediction likelihoods of CNVseq-AOH using 0.1-fold depth. The higher the confidence, the closer it is to 1, indicating that the region we predicted is likely to be AOH regions; **e** Chromosome 6

Interestingly, we found an AOH region (seq[GRCh38] hmz(6)(p12.3q12) chr6:g. 47568317_64568317hmz) using CNVseq-AOH, which crossed the centromeric regions of chromosome 6 in this case (Fig. 3c, d). Although with sufficient markers for this region (Fig. 3a), no AOH was reported in this region by CMA, which indirectly reflects the detection performance of CNVseq-AOH for regions crossing the centromeric regions. This AOH was further validated using high-coverage data, which also showed positive signals in this region (Fig. 3b).

### RNN VS. Hidden Markov model

**Fig. 4:**
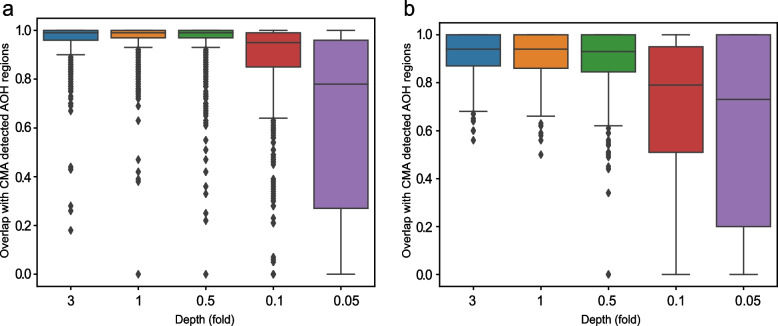
Method comparison for the 863 AOHs. **a** Performance of CNVseq-AOH; **b** Performance of HMM

RNN and Hidden Markov Model (HMM) are both widely used models for processing sequential data. HMM, a probabilistic model, is particularly effective for problems involving time series data. Currently, no published literature employs the HMM method for the detection of AOH, hence it cannot be cited. In this study, HMM with Gaussian emissions (the “hmmlearn.hmm.GaussianHMM” module in Python) was used for AOH prediction. We established an HMM model with 5 hidden states and a full covariance matrix, and compared it with CNVseq-AOH for AOH prediction. As a result, the prediction sensitivity of CNVseq-AOH is better than the HMM-based method with differing depths (Fig. 4).

### Validation of CNVseq-AOH with 6 clinical cases

We further applied CNVseq-AOH on 6 clinical cases with previously detected AOHs (Table 1). A mean depth of 0.573-fold (raw reads) was obtained for each sample. Uniquely aligned high-quality reads (UAHRs) reads were used for the detection of AOH. A UAHR was defined as a read that was uniquely aligned to the human genome reference with a quality value of more than 20 per base (containing no partial adapter sequences and no more than 5% that were not determined in the read length).

**Table 1:** Validation of CNVseq-AOH with 6 clinical cases

Case	Phenotype	Previous testing method		CNVseq-AOH		
		**Previous testing method**	**AOH region**	**AOH region**	**Sequencing depth (fold)**	**Overlap with WES detected AOH (100%)**
Case 1	HP:0001263 HP:0008850	WES	chr15:24,585,666–28,174,351	chr15:25,746,350–89066350	0.41	67.66
			chr15:33,407,799–86156769			100
Case 2	HP:0001511 HP:0008897 HP:0004322 HP:0011968 HP:0001511	WES	chr7:7,260,369–8660370	chr7:2,251,858–29,851,858 chr7:49,571,858–58,021,858 chr7:60,921,858–100821858 chr7:129,931,858–157,701,858	0.49	100
			chr7:10,360,373–27760381			100
			chr7:62,639,622–71,935,015			100
			chr7:74,585,671–81,070,684			100
			chr7:84,370,684–97570688			100
			chr7:132,315,241–139,115,254			100
			chr7:154,608,290–159207311			67.26
Case 3	HP:0000510 HP:0000662	WES	chr8:53,487,440–90487772	chr8:53,035,816–91755816	0.6	100
Case 4	HP:0001511 HP:0001324 HP:0011968 HP:0001197	WES	chr15:23,440,748–28234345 chr15:75,707,659–91856770	chr15:23,663,671–31,173,671 chr15:74,433,671–93,573,671	0.7	95.35
						100
Case 5	HP:0001655 HP:0000951 HP:0000035 HP:0001622 HP:0001561	WES	chr15:24,582,838–28,174,600 chr15:34,807,799–101759797	chr15:24,898,545–100,268,545	0.5	91.21
						97.77
Case 6	HP:0001270 HP:0012758	WES	chr15:23,445,182–39,020,912	chr15:23,593,671–37,083,671	0.74	86.61

As a result, CNVseq-AOH detected all the 15 AOH regions (Table 1). In some cases with multiple known AOHs (Case 1, Case 2, and Case 5), a greater number of AOH regions were detected by CMA, probably because several AOH regions were split into sub-regions by CMA.

## Discussion

RNN, known as recurrent neural network, is a very popular class of neural network. RNN is especially useful with sequential data. The neuron in RNN can use the internal state to “memory” previous input information, combining the information of the current input, to determine the next output state. RNN was widely used in natural language processing (NLP) [21]. However, the application of RNN in human genomic research is still rare. In this study, we described an RNN-based method, CNVseq-AOH, for predicting the absence of heterozygosity using LP-WGS. To the best of our knowledge, CNVseq-AOH is the first application combining population-based haplotype information, adjustable sliding windows, and RNN in genetic testing. CNVseq-AOH shows the feasibility of using ultra-low sequencing depth for the detection of clinically significant AOHs and demonstrates its potential in genetic testing.

One of the key innovations of CNVseq-AOH is the use of population-based haplotype information. Based on our testing, population-based haplotype information greatly influenced the feasibility of CNVseq-AOH. For the 409 samples in the current study, ancestry-matched populations (or genetically similar population) were used for analysis. We further compared the sensitivity using ancestry-matched populations for feature learning and using all available haplotype information from multiple ethnicities for feature learning at 0.1-fold. As a result, using a threshold of 50% overlap with the AOHs detected by CMA, the sensitivity of CNVseq-AOH reached 93.40% (806/863) when using ancestry-matched populations for feature learning. When switching to the strategy using all available haplotype information from multiple ethnicities for feature learning, the sensitivity is only 60.95% (526/863). Simultaneously, when employing a strategy using all available haplotype information from multiple ethnicities for feature learning, the accuracy is also significantly impacted (Supplementary Fig. 3). Not all the populations are captured in the 1KGP. The number of samples in a specific population varied a lot. This may influence the accuracy of our method and impede the wide application of CNVseq-AOH. Expanding the data collection to include new populations and samples may solve the problem.

One limitation of CNVseq-AOH is that it cannot be used for the detection of mosaic AOH. So, we did not include the 4 cases with mosaic AOH for testing in the first place. Based on the signals for these 4 cases (Supplementary Fig. 4), CNVseq-AOH possesses the potential for predicting mosaic AOH. This may require a different model and a great number of ascertained positive cases with mosaic AOHs for model training, which is an interesting topic but beyond the scope of this study. Another limitation of the current study is the performance of CNVseq-AOH for the detection of AOHs on the X chromosome. With a depth of 0.1-fold, a detection sensitivity of only 59.49% was achieved for the 79 AOHs on the X chromosome in the 1KGP. In phase three of the 1KGP, variants on the X chromosome were phased without the pedigree-based correction using Eagle2 (http://ftp.1000genomes.ebi.ac.uk/vol1/ftp/data_collections/1000G_2504_high_coverage/working/20201028_3202_phased/README_SNV_INDEL_phasing_111822.pdf), resulting in less number of SNPs per 1 Mb. So, the information of biallelic SNPs in the VCF file for the X chromosome is insufficient to calculate the probabilities for resampled reads. Actually, this is not a limitation of CNVseq-AOH, which means that, with sufficient information in the reference panel, CNVseq-AOH also possesses the potential to provide high prediction sensitivity for AOHs located on the X chromosome. Next, we plan to reanalyze these samples to optimize the performance of CNVseq-AOH for the detection of AOHs on the X chromosome.

In this study, we investigated sequencing depth on model performance in the 1KGP. In general, the prediction sensitivity of CNVseq-AOH increased with sequencing depth. However, data in the 1KGP was generated using various sequencing parameters (different sample types, library construction protocols, sequencing platforms, etc.), so the evaluation of sequencing depth may be biased. For clinical laboratories, depth evaluation using real clinical samples and uniform sequencing parameters is necessary before clinical application.

## Conclusions

In summary, we developed a method for predicting the absence of heterozygosity using LP-WGS data, which overcomes the sparse nature of typical LP-WGS by combing population-based haplotype information, adjustable sliding windows, and RNN. Next, we plan to apply our method to clinical pregnant women who underwent prenatal diagnosis, thereby further evaluating the performance and potential utility of CNVseq-AOH under realistic clinical scenarios.

### Supplementary Information

Supplementary file 1.

Supplementary file 2.

## Data Availability

The raw data of the cases with previously identified AOH events based on SNP-based microarrays and high coverage WGS data from 1KGP is available in the (https://www.ebi.ac.uk/ena/browser/view/PRJEB31736?show=reads) under the accession number PRJEB31736. The data of the 6 clinical cases generated and analyzed during the current study is not publicly available as they are patient samples and sharing them could compromise research participant privacy.
