# Supplementary material for: Recurrent neural network for predicting absence of heterozygosity from low pass WGS with ultra-low depth
Source: BMC Genomics. 2024 May 14;25:470. doi: 10.1186/s12864-024-10400-4 (PMC11092001; doi:10.1186/s12864-024-10400-4)

**Supplementary Figures**

**Supplementary Figure 1** Length distribution of additional AOHs detected by CNVseq-AOH with 0.1-fold depth in the 1KGP


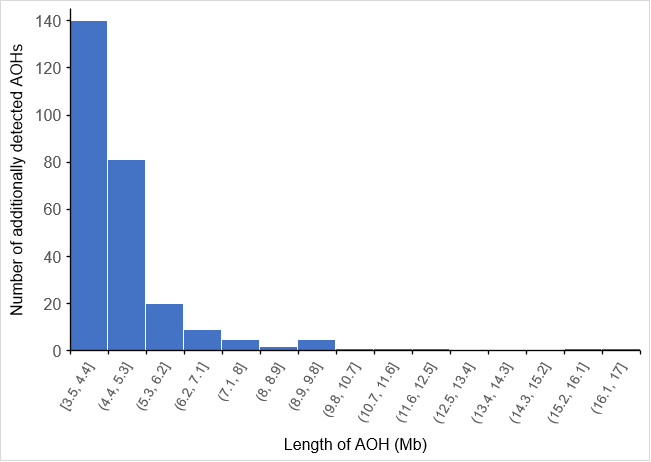


**Supplementary Figure 2** Regarding the additional AOHs detected by CNVSeq-AOH with a depth of 0.1-fold, 50.56% (135/267) additional AOHs were true positives by visualization using an in-house script. Here, the results of visual verification for additional AOHs detected by CNVSeq-AOH in 4 representative cases were displayed.

HG01951 (chr7:82502038-93172038)


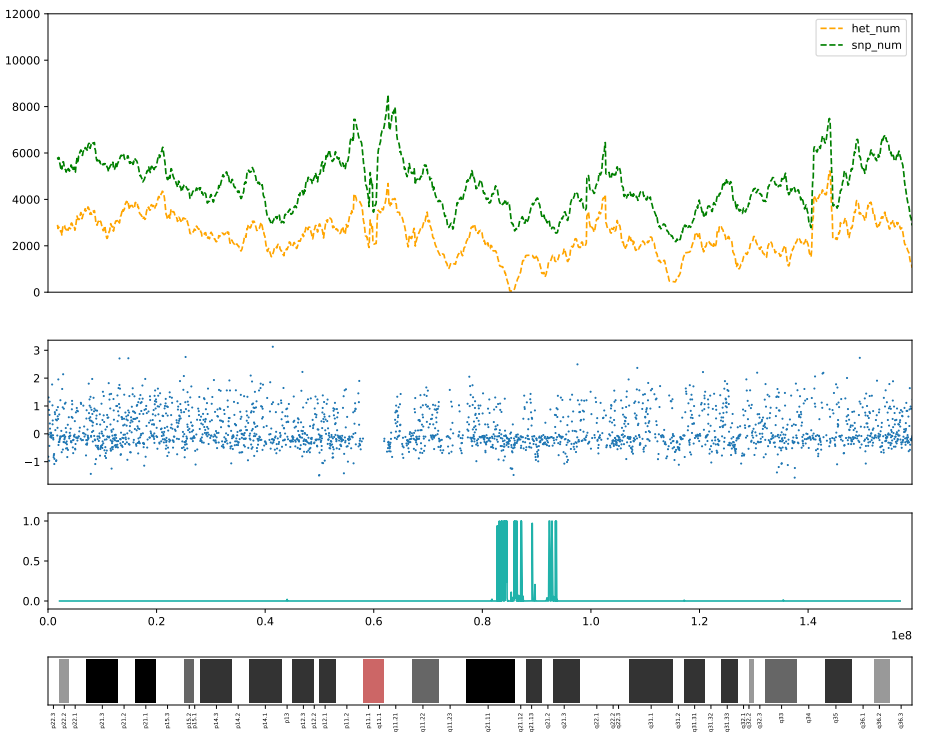


HG01497 (chr12:106600808-115120808)


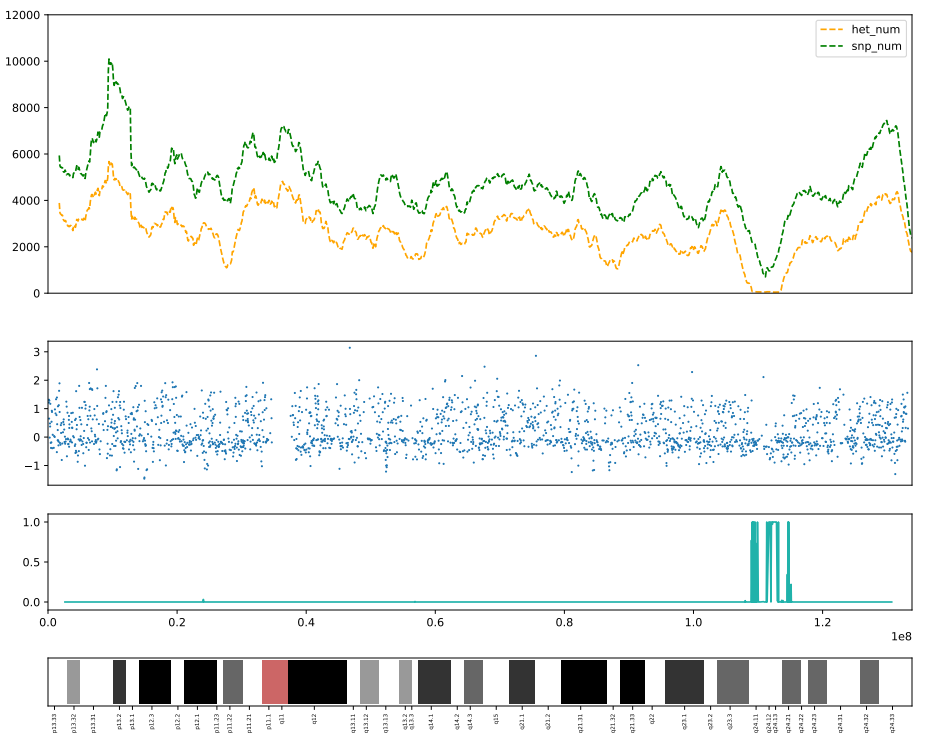


HG01348 (chr14:24868398-32248398)


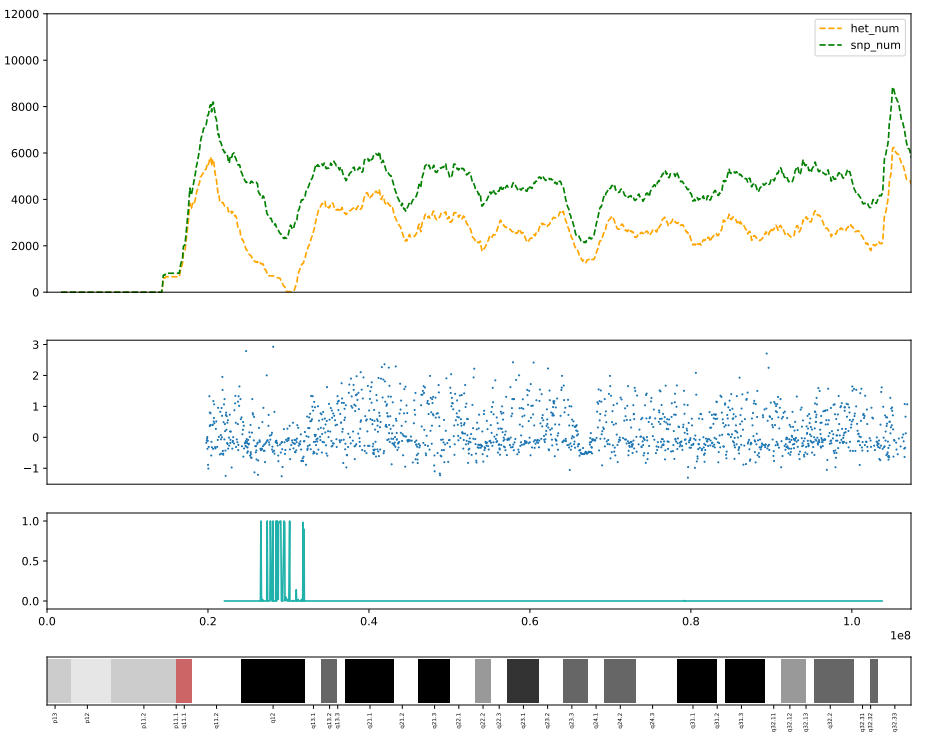


NA21108 (chr16:5695629-10565629)


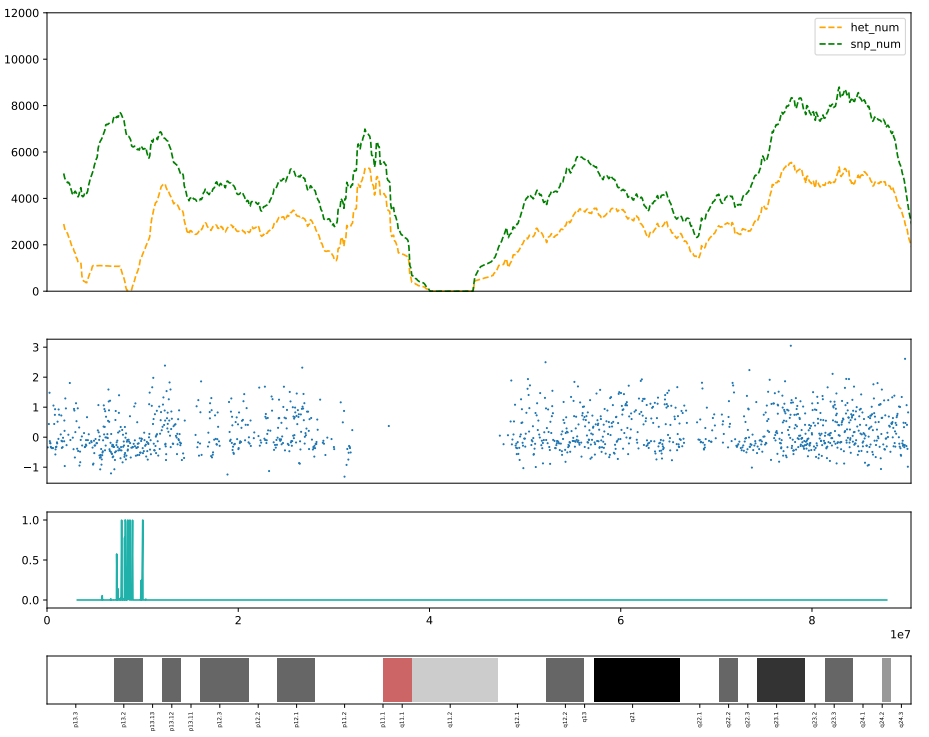


**Supplementary Figure 3** Comparison of the two strategies for the 409 samples (a. the strategy using ancestry-matched population; b. the strategy using all available haplotype information from multiple ethnicities)


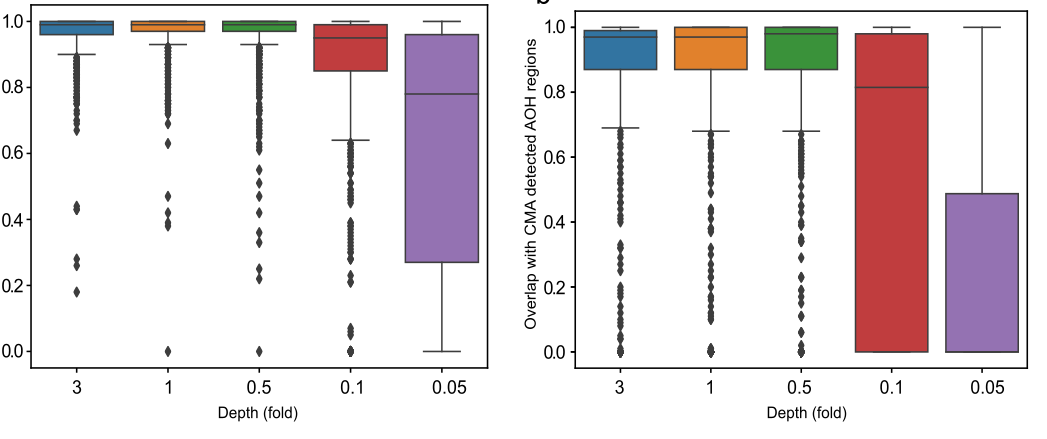


**Supplementary Figure 4** SNP ratio for the 4 cases with mosaic AOH using 3-fold depth


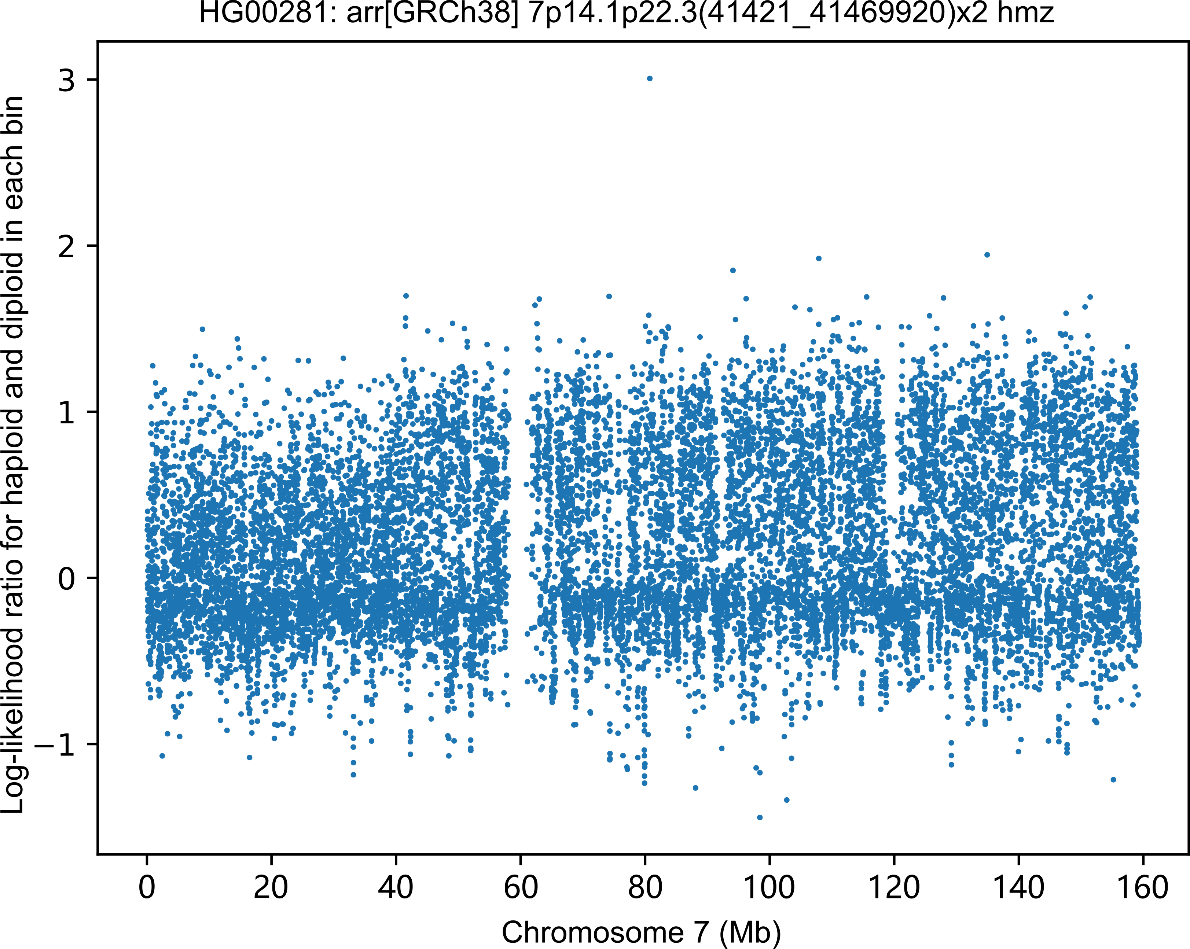


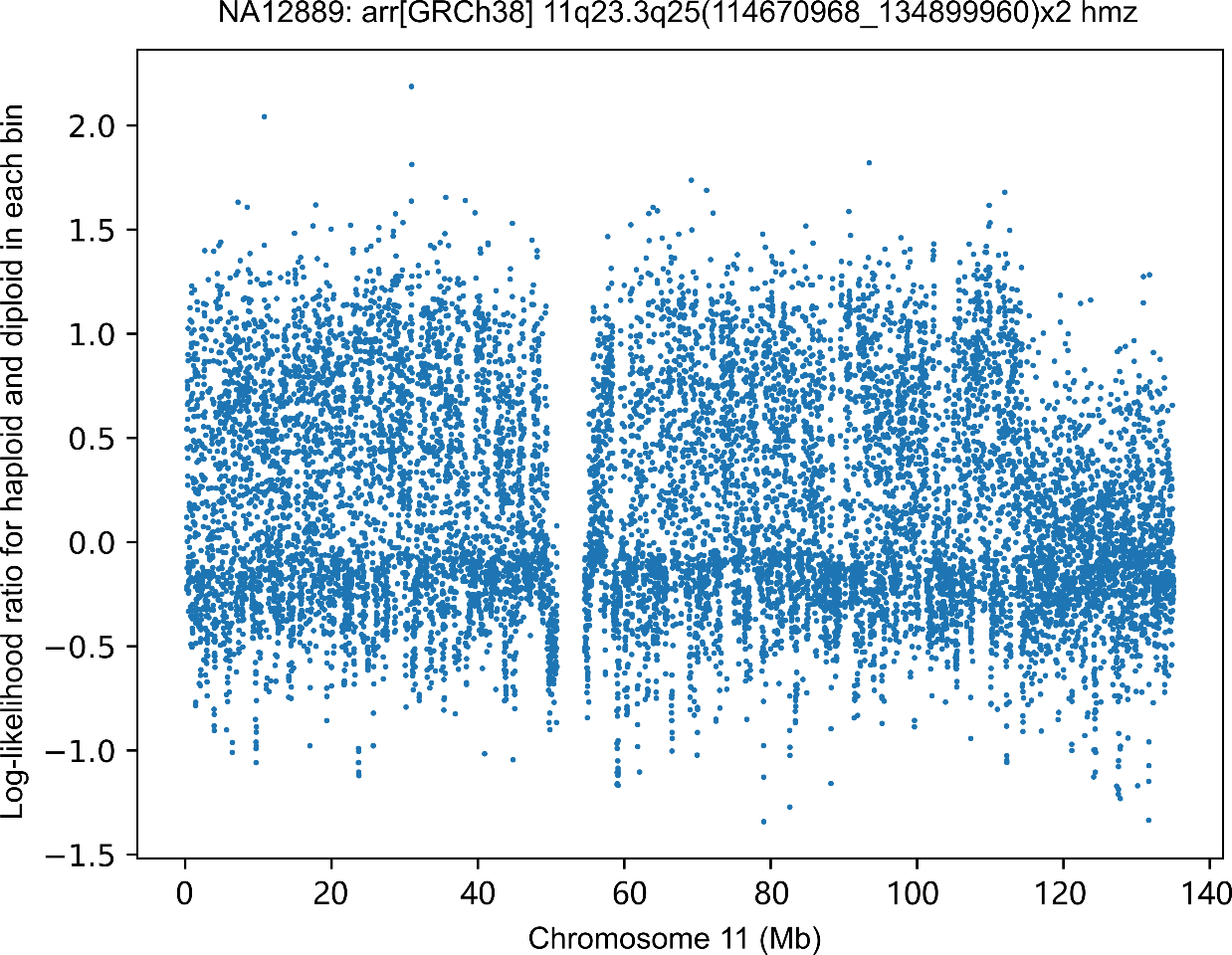


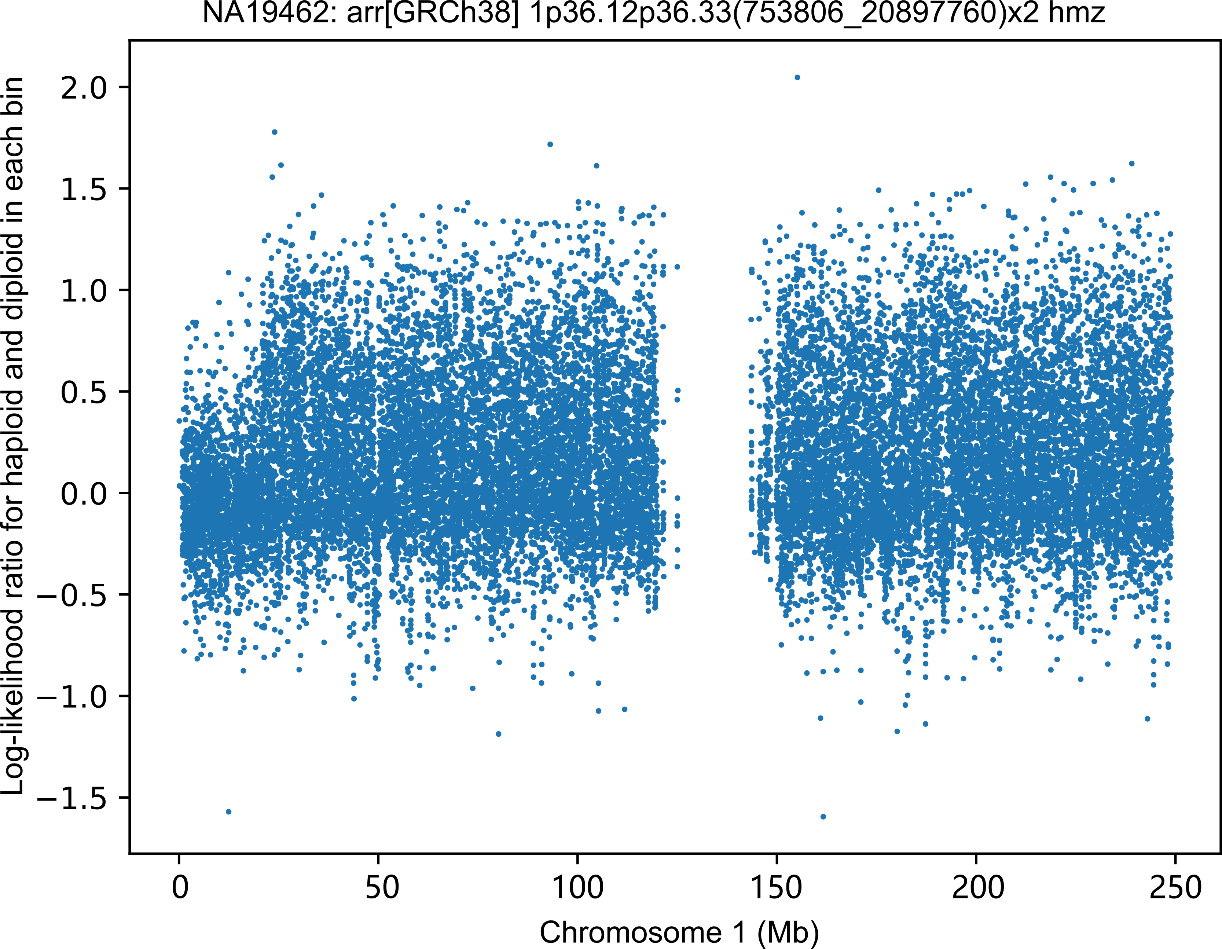


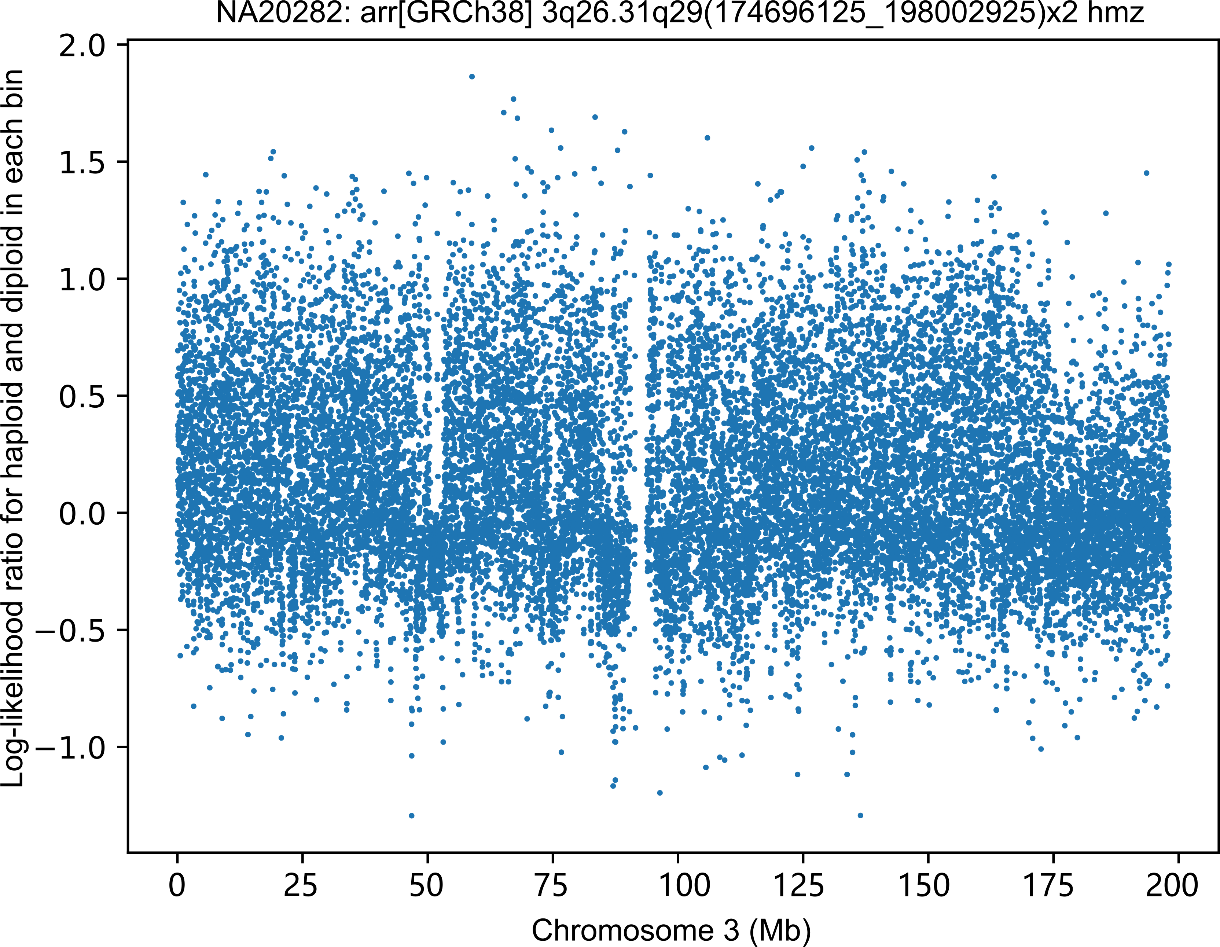

Supplement: Supplementary file 1 [file 12864_2024_10400_MOESM1_ESM.docx]
